# Mapping the Organization of Axis of Motion Selective Features in Human Area MT Using High-Field fMRI

**DOI:** 10.1371/journal.pone.0028716

**Published:** 2011-12-07

**Authors:** Jan Zimmermann, Rainer Goebel, Federico De Martino, Pierre-Francois van de Moortele, David Feinberg, Gregor Adriany, Denis Chaimow, Amir Shmuel, Kamil Uğurbil, Essa Yacoub

**Affiliations:** 1 Department of Cognitive Neuroscience, Faculty of Psychology and Neuroscience, Maastricht University, Maastricht, The Netherlands; 2 Maastricht Brain Imaging Center (M-BIC), Maastricht University, Maastricht, The Netherlands; 3 Department of Neuroimaging and Neuromodeling, Netherlands Institute for Neuroscience, an Institute of the Royal Netherlands Academy of Arts and Sciences (KNAW), Amsterdam, The Netherlands; 4 Department of Radiology, Center for Magnetic Resonance Research, University of Minnesota Medical School, Minneapolis, Minnesota, United States of America; 5 Advanced MRI Technologies, Sebastopol, California, United States of America; 6 Helen Wills Institute for Neuroscience, University of California, Berkeley, California, United States of America; 7 Department of Radiology, University of California San Francisco, San Francisco, California, United States of America; 8 Max Planck Institute for Biological Cybernetics, Tübingen, Germany; 9 Montreal Neurological Institute, McGill University, Montreal, Quebec, Canada; University of Leuven, Belgium

## Abstract

Functional magnetic resonance imaging (fMRI) at high magnetic fields has made it possible to investigate the columnar organization of the human brain in vivo with high degrees of accuracy and sensitivity. Until now, these results have been limited to the organization principles of early visual cortex (V1). While the middle temporal area (MT) has been the first identified extra-striate visual area shown to exhibit a columnar organization in monkeys, evidence of MT's columnar response properties and topographic layout in humans has remained elusive. Research using various approaches suggests similar response properties as in monkeys but failed to provide direct evidence for direction or axis of motion selectivity in human area MT. By combining state of the art pulse sequence design, high spatial resolution in all three dimensions (0.8 mm isotropic), optimized coil design, ultrahigh field magnets (7 Tesla) and novel high resolution cortical grid sampling analysis tools, we provide the first direct evidence for large-scale axis of motion selective feature organization in human area MT closely matching predictions from topographic columnar-level simulations.

## Introduction

Vision is the human's primary interface to the outside world. To build a coherent percept, the visual system must computationally solve the problem of recognizing objects in a dynamic environment. It is, thus, essential for the brain to analyze the information that is carried by the displacement of objects as well as the displacement of the observer, ultimately leading to the perception of motion. The processing of this information is likely to involve neuronal ensembles at the cortical column level [1], [2], [3], [4] as proposed for the middle temporal visual area (MT or V5).

In penetrations of single cell electrode recordings, neuronal response properties in many cortical areas remain relatively constant as one moves perpendicular to the surface of the cortex, while they vary in a direction parallel to the cortex. Such columnar organization is particularly evident in the visual system subdividing cortical territory in elementary units of operation, consisting of clusters of neurons sharing similar functional properties; in retinotopically organized visual areas cortical columns with the same feature selectivity are repeated several times each responding to different parts of the visual field. Columnar organizations have been investigated in great detail using electrophysiology [5], [6] and optical imaging [7], [8] in animals. The superior temporal sulcus (STS) of the macaque brain contains a multitude of areas that have been found to be selective to visual motion [9], [10], [11]. One of the most extensively studied areas in the macaque's brain is MT. The overall MT complex was first discovered by Dubner & Zeki (1971) [3] while recording single cell responses in the anesthetized macaque; they observed a distinct organization of cells that were direction of motion selective. While they proposed MT to be the first extrastriate area found to exhibit a columnar feature organization of direction selective neurons (DSN), corroborating evidence only came more than 10 years later [1], [2]. The columnar organization found in MT consists of smooth changes in the preference of neurons for direction of motion while occasionally being interrupted by opposing direction preference jumps of about 180° [2], [12]. The smooth varying gradients of direction columns run side by side with columns tuned in the opposite direction and both extend through several cortical layers [2], [4], [13]. Aggregated functional clusters responding to opposing motion directions are referred to as *axis of motion* columns.

The first demonstration of a human MT area (hMT) was established using functional magnetic resonance imaging (fMRI) [14] and positron emission tomography (PET) [14] based on its stronger responses to moving compared to stationary stimuli. Subsequently, mapping motion selective visual areas as well as separating the hMT+ complex into its subcomponents MT, MSTd and MSTv has become standard practice using fMRI in both monkeys [15], [16] and humans [17], [18]. MT can be distinguished from MSTd on the basis of its exclusive response to motion in the contralateral visual field while the latter also responds to ipsilateral motion as well as complex flow patterns [17], [19]. Similarities between the human and non-human primate motion processing system support the notion that human MT would possess the same functional direction selectivity with analogous functional cortical organization. Previous attempts, using various approaches like fMRI adaptation experiments [20], [21], [22], multivariate pattern analysis [23] and pattern integration through complex motion analysis [24], [25] have provided indirect evidence for direction selective responses in human MT [26]. However, to date, *no* direct information exists about the spatial organization of such responses in humans, presumably because most commonly used fMRI approaches are incapable of columnar level spatial resolution [27] and specificity.

Recent advances in MRI technology, particularly the introduction and development of high magnetic fields [27] and imaging sequences, have led to improved spatial specificity [27], [28], [29] of functional mapping signals along with increases in both the signal-to-noise (SNR) [30] and contrast-to-noise (CNR) ratios in fMRI. These have enabled efforts aimed at imaging functional properties and organizations at the level of cortical columns [31], [32], [33], [34], [35], with particular success at the ultrahigh field of 7 Tesla (7T) [31], [32]. With increasing magnetic fields, the blood signal contribution from larger draining veins, which have poor functional specificity, is reduced compared to lower fields [27], while functional signals originating from smaller vessels, including capillaries, are sufficiently enhanced to become robustly detectable [28]. Further gains in functional mapping accuracy can be attained with the use of spin echo rather than the conventionally employed gradient echo (GE) fMRI approach [27], [29], especially at 7T and at higher fields, permitting reliable mapping of columnar organizations in the human brain [31], [32].

Despite these advances, the only columnar functional organization identified non-invasively in humans to date are ocular dominance [31], [35], temporal frequency [36] and orientation domains [32], all of which reside in the primary visual cortex V1. A key reason for this is the feasibility of using a single, several millimeters thick, 2D slice acquisition with submillimeter in-plane resolution to cover the anatomically identifiable calcarine sulcus. This approach is however, inadequate for mapping columnar organizations in higher level areas that possess more complex cortical folding and requires volumetric acquisition with *isotropic* sub-millimeter resolution. Furthermore, the investigation of complex cortical map structures needs advanced sampling to take into account the curvature and thickness of the cortical gray matter at every sampled data point.

An estimate of columnar organization in human MT is not available. However, organizational parameters can be estimated for the human MT based on previous work in animal models using invasive methods. Studies in cebus apella found the cycle length of axis of motion columns in MT to be around 1–1.4 mm [4]; in the same species, the width of single ocular dominance columns were reported to be approximately 350 µm [37]. Human work on ocular dominance columns (ODC) has revealed an approximate width of 1 mm [31], [38], [39] indicating a scaling factor of 2.8 from cebus apella to human columnar organization. Using the ODC scaling factor would predict the cycle length for axis of motion columns to be in the range of 2.8–4 mm. Considering the scaling factor for the ocular dominance and axis of motion columns from a new world monkey, of course, may not be the same; however, even a conservative two-fold scaling factor, which would put the cycle dimensions for the human axis of motion in the ∼2 to 2.8 mm range, would suggest that these columns are well within reach of sub-millimeter ultrahigh field fMRI approaches.

In this work, using ultrahigh field (7T) fMRI, we provide direct evidence of the existence of axis of motion selective features in human area MT and in addition present spatial maps depicting their columnar organization. To do so we employed, for the first time, a three dimensional, imaging sequence (3D GRASE with inner volume selection) [40], [41], [42] that incorporates functionally more accurate spin [27], [29] and stimulated [43] echo based contrast, and a unique analysis approach based on topographical mapping, yielding information on the organization principle of axis of motion columns in human MT. Further, we demonstrate by means of data analysis and computational modeling, that these methods provide the necessary sensitivity, spatial resolution, and volume coverage to resolve the fine grained organization of feature representations within higher level, folded cortical areas.

## Results

Three subjects underwent high field 7T fMRI to localize motion selective area hMT. After localizing the hMT area, and distinguishing it from other subfields of the hMT+ complex, using a conventional gradient echo fMRI acquisition, in a separate scanning session, a reduced FOV, inner volume selective (or “zoomed”) 3D GRASE sequence [40], [41] was employed with sub-millimeter spatial resolution (0.8 mm isotropic) to investigate axis of motion selective features in hMT at columnar resolution. Mapping of motion direction responses was performed by presenting moving random dot patterns in one of 8 directions using a slow event related paradigm. These 8 separate directions were reduced to 4 distinct sets, by combining two opposing motion directions, resulting in data representing axis of motion selectivity (see data analysis). Modeling of columnar organization reconstruction followed procedures described in Chaimow et al. [44] adapted to the orientation model of Niebur and Wörgötter [45]. Columnar width was modeled liberally, using a 4 mm cycle, and conservatively using 2 mm as well as 1.4 mm cycles and a BOLD point spread function of 1 mm FWHM [28] (see introduction as well as experimental procedures).

Results of the hMT localization and separation from hMST are shown in Fig. S1. Area hMT is identified in two subjects' left (S1 & S3) and one subject's right (S2) superior temporal sulcus closely following the individual's white-gray matter boundary. Given the limited imaging volume of the high resolution 3D GRASE acquisition, only a single hemisphere could be fully optimized with the slice prescription. The regions identified as hMT lie within the posterior part of the superior temporal sulcus in close resemblance with the results of previous localization studies [17], [18]. The number of voxels defining the hMT area in the cortical gray matter was approximately 500 (S1 left hMT, number of voxels = 448; S2 right hMT, number of voxels = 426; S3 left hMT, number of voxels = 469). Fig. S1 depicts the average fMRI time-course for left hMT of subject S1 in one localizer run. Results clearly demonstrate the high selectivity obtained for localization. For all subjects, voxels selected exhibited a significantly larger response (p<10^−4^, whole brain, FDR corrected) to moving compared to stationary stimuli presented in the contralateral as well as simultaneously both visual hemifields. Responses to moving stimuli presented in the ipsilateral hemifield did not differ significantly from their respective static counterpart as expected. To additionally confirm the localization and separation of MT from its motion selective satellites, standard retinotopic procedures in two subjects (S1 & S3) confirmed the selected ROI's restricted selectivity by representing the complete contralateral hemifield (fig. S8).

**Figure 1:**
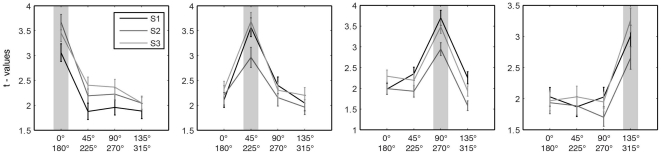
Axis of motion selectivity tuning for all three analyzed subjects. Tuning curves were obtained by 500 fold cross validation (25% leave out) and averaging the response properties (*t* values of standard GLM) of all voxels sharing the same best axis of motion fit in the motion direction experiment. The plots depict the mean and standard error across folds of all voxel sharing the same maximum fit (grey highlighting) on a single subject level.

In the motion direction mapping, all voxels within each subject's hMT ROI exhibited a significant response to moving stimuli (p<10^−4^, Bonferroni corrected). Restriction of activation to hMT in the spin-echo data is shown as an example for subject S1 in figure S2. Figure 1 depicts the tuning characteristics of all voxels (n = 1343) sharing the same preferred axis of motion fit for all analyzed subjects. A characteristic peak at the preferred axis of motion can be observed in all subjects showing that the averaged voxel responses exhibit a clear preference towards a single axis of motion. These tuning curves were obtained from the voxels in the region identified as hMT in each subject, without further resampling as performed for generation of the surface-based functional maps (see below). The distribution of voxels characterizing one specific axis of motion differed significantly from an equal distribution in subject S1 but not in the remaining two (χ^2^_3_ S1 = 28,45, p<0.01; χ^2^_3_ S2 = 5,49, p>0.1; χ^2^_3_ S3 = 5,52, p>0.1). These findings are in agreement with the early electrophysiological work [2] in which the number of neurons preferring a specific motion direction in macaque MT was found to be variable, however, without a clear bias towards any particular direction.

**Figure 2:**
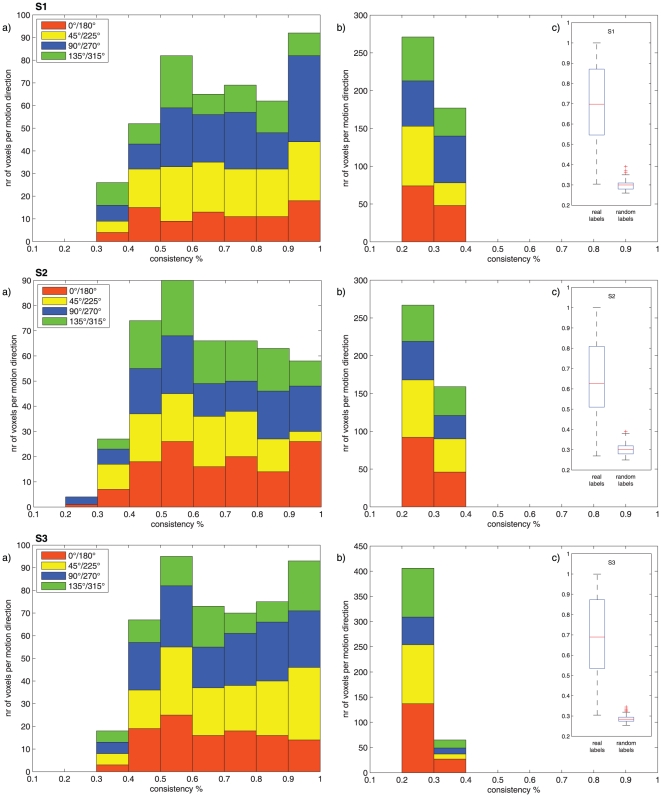
Consistency plots for the cross validation and permutation tests for all three analyzed subjects (25% leave out). Consistency distributions using real (a) and random (b) labels show the highest consistent axis of motion preference for every voxel in 500 permutations. (c) Comparison of real and random labels depicts the highly above chance consistency of most voxels analyzed (92.5% of voxels above chance, over all three subjects).

To investigate the overall consistency of voxel tuning properties we used a cross validation procedure (see experimental procedures). Figure 2a depicts the overall consistencies of all voxels in the three analyzed subjects. In general, the majority of voxels (consistency values 0.4 and higher, p<0.01) show a high degree of consistency. No bias in the distribution of axis of motion tuning can be observed. Permutation testing (see experimental procedures) was used, on the individual datasets, to evaluate the significance of these results. Figure 2b depicts the empirically estimated null distribution for all subjects. A comparison of the two distributions between real and random labels can be seen in Figure 2c. Of all 1343 analyzed voxels in our subjects, only 8.7% (115 voxels) fall within the chance level consistency distribution. The vast majority of voxels, therefore, have a significant consistency of the labeled axis of motion.

**Figure 3:**
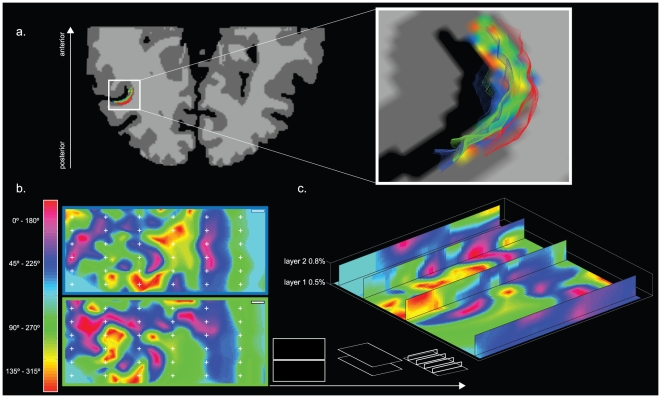
Illustration representing the axis of motion columnar mapping approach exemplary in subject S1. (a) Results of MT localization are projected onto the cortical reconstruction of the subjects left hemisphere (neurological convention). High resolution cortical grid sampling is performed. Zoomed in view to the subjects STS with overlaid streamlines at two relative cortical depths (0.5, and 0.8%). In order to not suffer from residual contribution from superficial blood vessels, sampling was restricted to the mid-level and deeper layers (b) Results of the high resolution cortical grid sampling for the motion direction experiment showing columnar organization of axis of motion features in two sampled layers (scale bar = 1 mm, color frames representing the streamlines in zoomed in view). (c) Representation of the high resolution cortical grid sampling showing four three dimensional vertical slices through the sampled cortical layers depicting the consistency of cortical columns tangential to the surface.

Functional maps, with voxels labeled for preferred axis of motion, were created in volume space for each subject. This was performed using a novel cortical depth sampling approach (see methods) that is based on preprocessing steps used in cortical thickness measurements [46]. To sample the co-registered individual axis of motion maps, high resolution cortical grids were created at two depth planes covering the entire subject's hMT ROI; in order to obtain smooth curvature boundaries, three dimensional upsampling with 10 data points per actual voxel was used (Fig. 3a). Figure 3b shows the flattened results of the high resolution cortical grid sampling for subject S1 (for a comparison of flattened results from all subjects see Fig. S4) at two relative cortical depth levels (0.5, and 0.8 percent of 2.3 mm mean cortical thickness) with an area of 78.12 mm^2^ (12.4 mm anterior-posterior×6.3 mm inferior-superior). Due to the high resolution imaging employed, distinct voxels at both levels were given for most of the ROI. Visual observation reveals an arrangement of smooth varying gradients of axis of motion tuning within the cortical plane that is, in terms of its arrangement characteristics, very similar with the findings from monkey cell recordings [2], [4] as well as our simulations results (see below). Importantly, this topographical columnar organization can be observed in all sampled planes at a given cortical depth (Fig. 3b). The reliability of the produced axis of motion maps was computed following the same cross validation procedure adopted for voxel wise measures of selectivity (see experimental procedures). The high degree of consistency between different splits of the data can be seen in figure S5, exemplary for subject S1. Additionally, across session consistency (data split into two half's) can be seen in figures S6 and S7.

The organization principle can also be viewed perpendicular to the cortex, as it traverses vertically along the sampled cortical layers (Fig. 3c); some regions display similar axis of motion preference across cortical depth while other parts of the maps show additional gradual vertical changes. These maps demonstrate a systematic organization of axis of motion tuning in human area MT that is comparable to the results of other columnar mapping approaches in both human [31], [32] and monkey [2], [4], [7] cortex.

**Figure 4:**
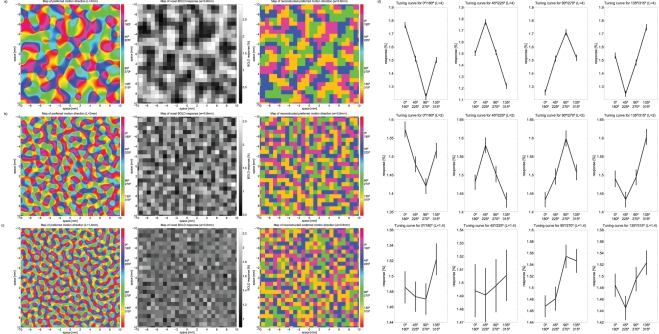
Simulation showing expected maps of axis of motion preference and tuning curves assuming isotropic columnar patterns both, liberal (4 mm, a), conservative (2 mm, b) and unrealistically small (1.4 mm, c) axis of motion cycle length. (a, b, c) Maps of axis of motion preference were modeled according to Niebur et al. (1993) (first column). The second column shows corresponding maps of responses to one axis of motion sampled by MRI voxels. Finally responses from 4 simulated axis of motion directions were used to obtain a simulation of estimated maps of preferred axis of motion (third column). In both realistic cases (2 mm cycle length and 4 mm cycle length) reconstructed axis of motion preferences resemble the original maps while in the unrealistic case (1.4 mm) resemblance starts to break down. (d) Results of simulated axis of motion selectivity tuning curves (compare to figure 1). Curves were computed from modeled voxel maps mirroring the process used to obtain empirical tuning curves by simulating a comparable noise level and a 500-fold cross validation. Error bars reflect the standard error of the mean across folds. For both, liberal (4 mm, first row), and conservative (2 mm, second row) axis of motion cycle lengths, tuning curves show robust axis of motion selectivity. For a cycle length of 1.4 mm no such selectivity can be observed.

In order to qualitatively compare our empirical findings with the expected topography, we performed a topographic columnar simulation based on Chaimow et al. [44]. The simulations provide a benchmark for the feasibility of columnar level mapping in light of our imaging parameters and the estimated axis of motion column cycle in the range of 2–4 mm. We modeled columnar organization reconstruction with a MT column cycle of 4 mm, 2 mm and 1.4 mm (see methods for details). The 2 mm cycle width represents a conservative estimate that assumes a scaling factor much smaller than the ODC scaling between cebus apella to human. The 1.4 mm cycle width, on the other hand is a lower bound that is unrealistic since it assumes that the capucine monkey and human columns are approximately the same size. Figure 4 shows the results of the simulations and their resulting image reconstructions for all cycle widths in addition to the corresponding tuning curves simulated using exact parameters from the original fMRI image acquisition. For both, liberal (4 mm) and conservative (2 mm) axis of motion cycle lengths, reconstruction closely resembles the underlying columnar organization principles, and corresponding tuning curves exhibit a clear differential response, that closely follows the experimental fMRI data. The variability (shown as vertical bars) observed in the 2 mm cycle width simulations approximates our experimental results (Fig. 1). Using an unrealistic cycle width of 1.4 mm, both preference reconstruction as well as tuning characteristics become unspecific and do not correspond with our findings.

## Discussion

The existence of direction selective neurons within area MT is a well-established feature in the functional organization of higher visual cortex. Studies using invasive electrophysiology [1], 2,3,4,47,48 or optical imaging [49], [50] have described the principle features of primate MT and cat area 18. The functional organization is composed of smooth varying gradients of columns containing neurons that react specifically to a certain motion direction. Those columns are found running side by side with their respective opposing motion direction counterparts. In aggregating opposing motion directions, larger axis of motion features can be constructed that are more easily detectable with ultra high-field fMRI than individual direction selective columns. The results presented in this paper are the first direct demonstration of axis of motion selectivity and tuning characteristics in the human brain extending previous studies [22], [23], [24], [51]. Furthermore, the results present the first direct functional mapping that provides insights into the spatial organization of human area MT for axis of motion selectivity.

Using ultrahigh field neuroimaging at 7 Tesla, coupled with optimized multichannel RF coils, and a 3D gradient echo and highly specific spin/stimulated echo weighted fMRI acquisition, we were able to sample the functional responses along the complex curvature of the superior temporal sulcus at a very high spatial resolution (0.512 mm^3^ nominal voxel volume). As in all sequences that achieve spatial encoding after a single excitation (such as EPI), spatial blurring beyond the nominal voxel dimensions is encountered in 3D GRASE. FOV reduction, as employed in this study and in our previous spin-echo based columnar mapping [31], [32], minimizes this significantly.

To our knowledge, this study is also unique in investigating the columnar level functional properties of an extrastriate area sampled in multiple layers. Our results reveal a clustered organization of axis of motion columns within 3 subjects and a reliable, clear organization principle that extends tangentially along the cortex. While our scanning resolution is not high enough to resolve direction-selective columns in hMT, it appears to be sufficient to reliably resolve large-scale axis of motion columns with an estimated size of at least 2 mm. The vertical organization of axis of motion columns, in the dimension traversing the cortical laminae, reveals some variability that could reflect multiple causes. Such variability, vertical to the cortical surface, were also reported for axis of motion columns by early mapping approaches using single cell penetrations [2] and thus, could be an expected property. However, because of the curvature of the cortical gray mater ribbon, the columns may not traverse the different lamina along lines perpendicular to the surface, getting obligatorily wider on the surface with the lower curvature [52]. In this case, the columns may not run perfectly aligned to the sampling direction across the layers, a problem that is encountered also in electrophysiology but due to uncertainties in angle of electrode penetration. Finally, our sampling at two relative depth levels could still be insufficient to capture the full arrangement of vertical columnar properties and should, therefore, be seen as an early approximation. The close resemblance of our findings to conclusions reached in single cell electrophysiology [2], [4] or optical imaging [49], [50] recordings in animal models, however, provides support for our observations.

Besides the functional topographic columnar level axis of motion maps, our results provide direct evidence for axis of motion features in human MT. Furthermore, following the initial critical attempts to successfully identify known columnar organization of the human brain [31], [32], [33], [36], [39], this study demonstrates the feasibility of investigating feature organizations in folded patches of the human cortex without a priori knowledge of their topographical layout. Importantly, our simulations show that the acquired image resolution, coupled with the low PSF due to selective spin echoes, is sufficient for the mapping of cortical columns in the order of 2–4 mm in width which, as previously discussed, is the expected range of human axis of motion columns based on previous animal models.

Studying cortical columns, or groups of neurons that share similar response properties, can be seen as an adequate approach in understanding the fundamental units of brain computation. Even though the responses of single neurons are made up of complex dynamics that lead in their interaction to specific properties, cortical columns resemble the smallest unit of the brain that shares similar, but not identical, representational characteristics. Details on the columnar organization, therefore, provide a basic differentiation in information processing performed in brain areas. The present work demonstrates that this scale is within the reach of fMRI techniques in the human brain.

## Methods

### Ethics Statement

Studies were approved by the institutional review board of the ‘Human Research Protection Program’ at the University of Minnesota. All subjects gave their written informed consent before participating in the study.

### Subjects

Five healthy volunteers (2 males, 3 females) without prior history of psychiatric or neurological illness as well as normal or corrected to normal visual participated in the study. Two subjects were removed from data analysis because of uncorrectable within-session head motion and corresponding image acquisition artifacts.

### Experimental design and stimuli

Visual stimuli were presented using video projector and mirror at the rear of the magnet. An additional mirror was attached to the coil above the subject's eyes, allowing them to view a screen behind the coil where the image was projected onto. Stimuli were presented using custom-built stimulation software (StimulGL). Subjects were instructed to fixate on a small red central fixation spot during the entire functional scanning procedure. Eye movements were not recorded during scanning procedures. All subjects participating in the study were trained and experienced at maintaining fixation for long periods of time. Scanning sessions began with a combined T1 weighted MPRAGE and proton density acquisition, allowing for subsequent unbiasing of signal intensity profiles [53]. Area MT was functionally defined based on its responses to stimuli alternating between moving and stationary dot patterns, following standard procedures [14], [20], [54]. Dots travelled away and towards the fixation point (speed = 1 pixel per frame, dot size = 12 pixel, number of dots = 70, black dots on gray background) for 18 seconds followed by a stationary dot display for 10 seconds (Fig. S3a). To distinguish MT from MST in the hMT+ complex, the same alternating stimuli were additionally presented restricted to either the left or right visual hemifield using the same parameters (Fig. S3b). Because receptive fields in MST are larger than in MT [12], [17], evoked activity from the ipsilateral hemifield can be used to distinguish these areas from each other. Left, right and both lateral localization stimuli, as well as their static counterparts were presented 8 times, blocked into two functional runs of 5 min. 52 s. each (176 volumes per run). Stimuli used in retinotopic mapping procedures (eccentricity and polar angle) followed standard protocols and are described elsewhere [55], [56]. Retinotopic mapping was performed to additionally confirm the selected regions localization to MT and separation from satellite motion selective regions. Motion direction preference was mapped by presenting dots moving coherently into one of eight (0°, 45°,…, 225°, 315°) randomly presented directions (Fig. S3c). Moving dot patterns (speed = 2 pixel per frame, dot size = 10 pixel, number of dots = 300, black dots on gray background) were presented for 6 seconds followed by a variable inter trial interval (ITI) of 9–12 seconds to reduce functional signal carry over effects. A total of 24 trials per motion direction were obtained by randomly presenting each motion direction 4 times in 6 consecutive runs in order to reduce motion artifacts within the single runs. Coherent dot motion was chosen to maximize activity elicited by a single direction [57].

### MRI Acquisition

The image acquisition was performed at the Center for Magnetic Resonance Research (Minneapolis, MN, USA) using a 90 cm bore 7 Tesla whole-body magnet (Magnex Scientific, Abingdon, UK) driven by a Siemens console (Siemens Medical Systems, Erlangen, Germany). The RF coil consisted of a custom 6 channel receive array with elements (6 cm diameter) distributed symmetrically along the right - left direction and a separate open half-volume quadrature transmit coil to provide uniform excitation in visual areas. Such a design is especially ideal for high resolution spin-echo based fMRI because of the need for a relatively uniform 180 degree refocusing pulse, high sensitivity in the areas of interest, and an open design to allow for presentation of visual stimuli over a large visual field of view. For functional imaging, two different sequences were employed in the separate scanning sessions. The localizer experiments as well as the retinotopic measurements were conducted using a standard single shot gradient echo (GE) EPI sequence (echo time (TE) = 15 ms, nominal flip angle (FA) = 86°, slices = 40,TR = 2000 ms) with a reduced field of view (FOV = 128×128 mm^2^) and a 88×88 matrix resulting a nominal resolution of 1.45×1.45×1.5 mm^3^. For the axis of motion mapping, we used a single-excitation, 3D gradient and spin echo (GRASE) sequence [40], [41] with restricted FOV achieved *via* inner volume selection [42]. To do this, following excitation of a 3D volume, a slab selective gradient is applied along the phase encode direction during the 180 degree refocusing pulses, limiting the FOV in the phase encode direction, prior to 3D EPI readouts ((echo time (TE) = 40 ms, TR = 2000 ms, FOV = 25.6×204.8×9.6 mm^3^, matrix: 32×256×12) yielding a nominal resolution of 0.8×0.8×0.8 mm^3^). Positioning of the 3D GRASE sequence's limited FOV (phase encode and slice directions) was optimally prescribed according to individual results of the localizer (Fig. S1). Slice placement covered functionally localized hMT optimally in one selected hemisphere of each subject. Before the high resolution 7T functional session, a T1 weighted magnetization prepared rapid acquisition gradient echo (3D-MPRAGE) (176 slices, FOV = 136×256 mm^2^, matrix = 136×256, voxel size = 1×1×1 mm^3^) anatomical dataset was acquired for visualization of the functional results. Because MR images exhibit large, undesirable signal intensity variations at high magnetic fields originating from heterogeneous RF coil profiles [53], [58], additional gradient echo proton density (GE-PD) images (176 slices, FOV = 136×256 mm^2^, matrix = 136×256, voxel size = 1×1×1 mm^3^) were acquired to compute a ratio image with the T1 dataset, reducing the influence of the bias field [53], [59]. To reduce the influence of motion, subjects employed a bite bar made out of a hydroplastic material (Tak Systems) that matched their dental impressions.

### Imaging data analysis

Functional and anatomical images were analyzed using BrainVoyager QX (Brain Innovation, Maastricht, The Netherlands) as well as custom code in MATLAB (The MATHWORKS Inc., Natick, MA, USA). Anatomical T1 images obtained at 7T in the same session as the fMRI data were corrected for bias field inhomogeneities by computing the ratio with the simultaneously obtained PD images [53]. Preprocessing of the functional data included interscan slice-time correction (only for gradient echo data, since spin-echo data were acquired in 3D during a single echo-train of ∼300 msec), 3D rigid body motion correction, high-pass filtering using a general linear model (GLM) Fourier basis set as well as temporal gaussian smoothing with a full width half maximum (FWHM) kernel of 2 data-points. Average motion parameters for all three subjects included in the resulting analysis were as follows: translation, 0.09 mm±0.11 mm, 0.05°±0.05° rotation (mean ± SD of average parameters). Anatomical T1 data was up-sampled to 0.8 mm isotropic resolution to match the resolution of the functional data. Functional runs were coregistered to the individual anatomical T1 scan using the scanners positional information followed by manual fine alignement. All statistical computations were performed on a single-subject level using a GLM with a linear predictor for each experimental condition convolved with a standard hemodynamic response function. Area hMT was defined using the localizer experiment by selecting a region in the subjects unilateral posterior STS that exhibited a significant (p<10^−4^, whole brain, FDR corrected) response to bilateral and contralateral moving dot patterns in contrast to respective static patterns. To distinguish this area from the subregion MST of the MT+ complex, voxels were selected that did not exhibit a significant response to ipsilateral moving dot patterns (Fig. S1). Standard retinotopic mapping analysis was used to confirm the selected ROI's restricted response to the entire contralateral hemifield (fig. S8) and exclude satellite motion selective regions [18]. Individual regions of interests (ROI) were used for all subsequent analysis steps. All voxels in the entailing ROIs of each subject exhibited a significant response to moving stimuli during the axis of motion mapping experiment (p<10^−4^, FDR corrected). For all subsequent analysis steps, the presented 8 motion directions were grouped into 4 pairs of opposing motion directions (axis of motion) to increase the presumed size of columnar-level features since in monkeys, opposing motion directions are known to be arranged adjacent to each other [2]. Furthermore, this grouping was performed to achieve a higher amount of trials per predictor as well as more reliable parameter estimates.

Mapping of axis of motion columnar organization within the selected MT ROI was performed by fitting a standard GLM to the respective voxel time-courses and extracting *t* values for every axis of motion. The predictor with the highest fit, representing the predictor with the highest stimulus-induced normalized fMRI response (*t* - values), determined the characteristic preferred motion direction for every voxel. Motion direction tuning curves were obtained by 500 fold cross validation (75% preference classification, 25% averaging of voxel responses) of the above procedure. Results from the cross validation were then averaged and plotted with their respective standard error representing variability across the computed folds.

To assess the consistency of the produced maps, cross validation and permutation statistics were computed. For each subject, standard GLM fitting and “voxel labeling” was computed 500 times on a randomly picked subset of the data. Subsets were created by randomly removing 2 trials of each predictor for each of the 6 consecutive runs. Thus, each iteration contained a randomly reduced dataset with a total of 36 trials per predictor (25% leave out). The consistency of each voxel can, therefore, be defined as the highest number of equal axis of motion preference labeling during all iterations divided by the amount of iterations. To additionally verify the resulting consistencies of map structures, random relabeling of the individual subjects data was performed and subsequently tested using the same iterative procedure.

In order to create the topographic maps at different cortical depths, cortical tissue was segmented along the inner (white matter) and outer (CSF) boundary. Following Laplace's equation [46], different “potential” (intensity) values were defined for the inner and outer grey matter boundary. Solutions of Laplace's partial differential equation results in a smooth transition of “voltages” (intensities) from one boundary to the other. Such a solution can be found simply by keeping the values at the boundaries fixed and by smoothing the “voltage” values in between (grey matter voxels). From the obtained smooth field, a gradient value can be calculated at each voxel. Integrating along these gradient values results in “field lines” or “streamlines” used in cortical thickness calculations. For sampling data from an individual's preference volume map, sampling was performed orthogonal to the gradient in two orthogonal directions resulting in regularly spaced grid sample points at any initialized relative depth value. If, for example, the initialized depth value is 0.5, one grid axis will be created along the first chosen direction that traces a curved line through the middle of grey matter; at regular intervals along this first line, additional orthogonal lines will be started in orthogonal direction providing regularly spaced grid points along the second axis at the same relative depth level. This procedure is performed for each desired relative depth level. As opposed to using irregularly spaced mesh vertices for depth calculation, this approach provides precise geometrical information about path length and surface area when following folded cortex at different relative depth levels. Furthermore, streamlines (created when following the gradient from one side of cortex to the other side) precisely relate corresponding points within sampled regular grids across multiple cortex depth planes.

The modeling approach used closely follows procedures described in Chaimow et al. [44]. An isotropic pattern of cortical columns was modeled according to Niebur et al. [45] using a spatial cycle length of either 1.4 mm, or 2 mm or 4 mm. Hypothetical neuronal responses to 4 equally spaced axis of motion stimuli were modeled by assuming cosine shaped tuning curves. The obtained neuronal response maps were used to calculate maps of BOLD responses (point spread FWHM = 1 mm, asymptotic response = 3%), which in turn were sampled into voxels (voxel size = 0.8 mm). The PSF estimate was based on the observations that in the human brain, the GE fMRI PSF was estimated to have an upper limit of 2 mm FWHM in regions devoid of large blood vessels [28], [60], while in the cat brain, the PSF at FWHM for SE and GE fMRI was shown to be ∼0.7 mm and 1.7 mm, respectively. Additional details can be found in Chaimow et al. [44]. Axis of motion preference maps were reconstructed by assigning each voxel the axis of motion to which its response was maximal.

To model the empirically derived tuning curves, voxel responses for two data splits (75% for preference estimation and 25% for averaging responses into tuning curves) were simulated by adding normally distributed noise to the modeled response maps for each axis of motion stimulus. The standard deviation of the noise was estimated from the functional data as the standard deviation of the baseline and divided by the square root of the number of volumes used to estimate the responses. From the set of maps corresponding to the 75% split each voxel was assigned its preferred axis of motion. Finally tuning curves were calculated by averaging responses from the set of maps corresponding to the 25% split according to stimulus axis of motion from all voxels with the same preferred axis of motion. This process was repeated 500 times, after which a mean tuning curve and corresponding standard errors were computed.

## Supporting Information

Figure S1 **Localization of hMT exemplified in subject S1.** Upper panel depicts the results of the localization procedure superimposed by the field of view (blue frame) of the axis of motion spin echo experiment (images in radiological convention). Lower panel shows the averaged time course of one localization scan for hMT in subject S1 as well as the corresponding stimuli used.
(TIF)

Figure S2 **Specificity of the spin-echo sequence used in the axis of motion experiment.** Results show the F-map (p<0.01, Bonferroni corrected) comparing the mean of all presented motion directions to baseline exemplary in subject S1. The activity elicited by the stimulation is confined to MT (overlay of the ROI defined from the localization experiment in light green) closely following the cortical gray matter boundary and early visual areas.
(TIF)

Figure S3 **Summary of the stimulation paradigm used.** (a) Bilateral moving (18 s) versus stationary (10 s) random dots localizing the hMT+ complex. (b) Ipsilateral moving (18 s) versus stationary (10 s) random dots identifying MST. Peripheral dot patterns were presented in either the left or right visual field while subjects remained fixating on the central fixation spot. (c) Motion direction mapping (6 s) interleaved by a variable inter trial interval. Dots travelled coherently into one 8 randomly presented motion directions (0°, 45°,…, 270°, 315°).
(TIF)

Figure S4 **Comparison of the high resolution cortical grid sampling for the motion direction experiment in all subjects.** Columnar organization of axis of motion extending through two sampled layers (scale bar = 1 mm, color frames representing cortical depth, blue and green rectangles, 0.5%, 0.8% cortical thickness respectively).
(EPS)

Figure S5 **Consistency map for the cross validation test for subject S1 (leave 25% out).** Consistency distribution is shown using a heat map representing the highest consistent axis of motion preference for every map point in 500 permutations. Central aspects of the map show extremely high consistencies, meaning that axis of motion classification as represented by the axis of motion maps is highly consistent across the permutations.
(EPS)

Figure S6 **Consistency of the high resolution cortical grid sampling for the motion direction experiment in all subjects across two post hoc created sessions in which the data was split into two halfs (scale bar = 1 mm, color frames representing cortical depth, blue and green rectangles, 0.5%, 0.8% cortical thickness respectively).**
(EPS)

Figure S7 **Consistency of the high resolution cortical grid sampling in all subjects across two sessions (Figure S6) computed as angular difference maps.** Maps from each session were subtracted from the reference map of the full data set and the angular difference in axis of motion preference was plotted for each session.
(EPS)

Figure S8 **Results for the polar angle mapping performed in two subjects.** White bar (see arrow) in the identified hMT region represents the horizontal meridian.
(EPS)
