# Supplementary figures and images for: Mapping the Organization of Axis of Motion Selective Features in Human Area MT Using High-Field fMRI
Source: PLoS One. 2011 Dec 7;6(12):e28716. doi: 10.1371/journal.pone.0028716 (PMC3233606; doi:10.1371/journal.pone.0028716)

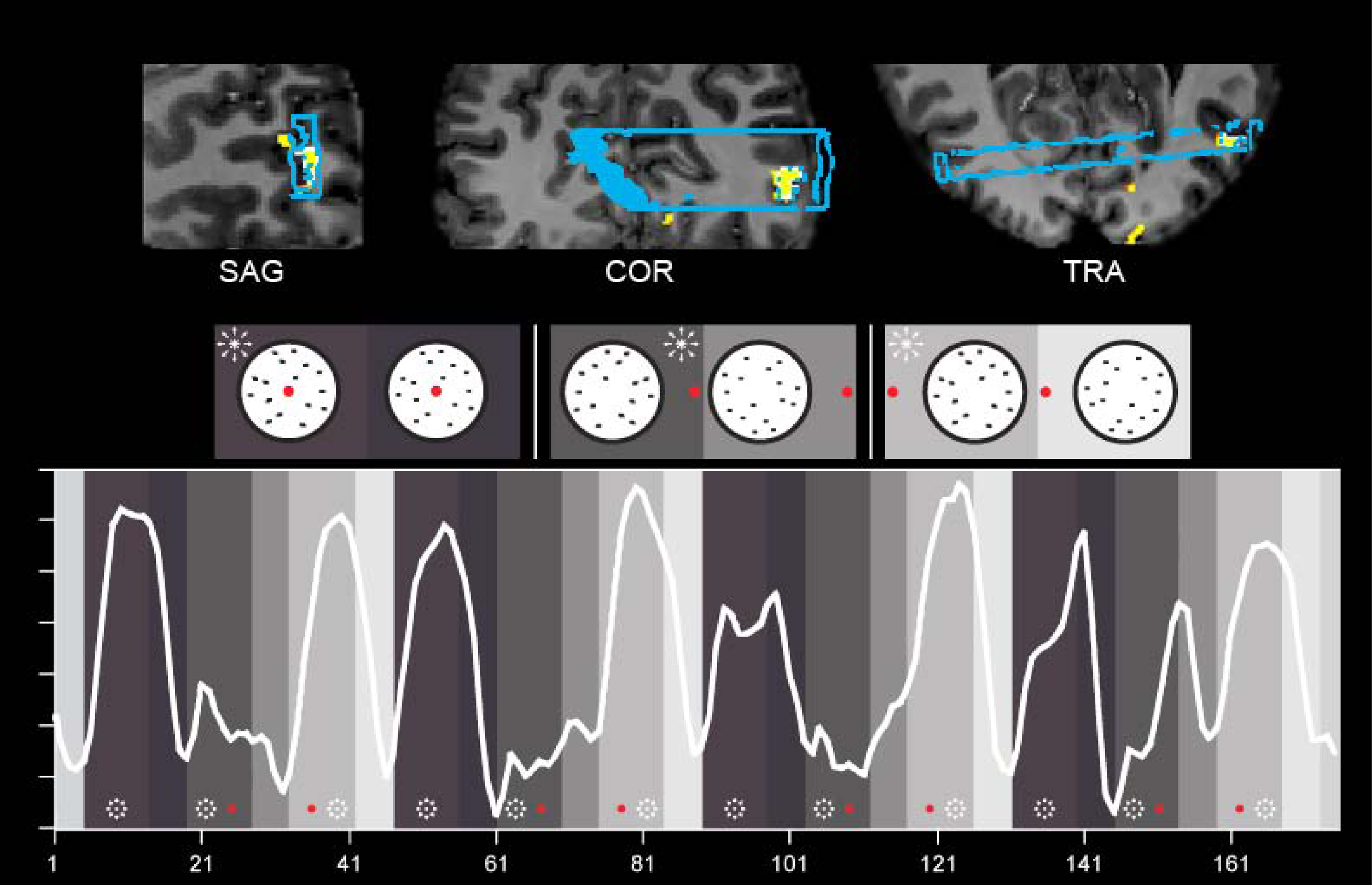

Supplement: Figure S1 — Localization of hMT exemplified in subject S1. Upper panel depicts the results of the localization procedure superimposed by the field of view (blue frame) of the axis of motion spin echo experiment (images in radiological convention). Lower panel shows the averaged time course of one localization scan for hMT in subject S1 as well as the corresponding stimuli used. (TIF) [file pone.0028716.s001.tif]

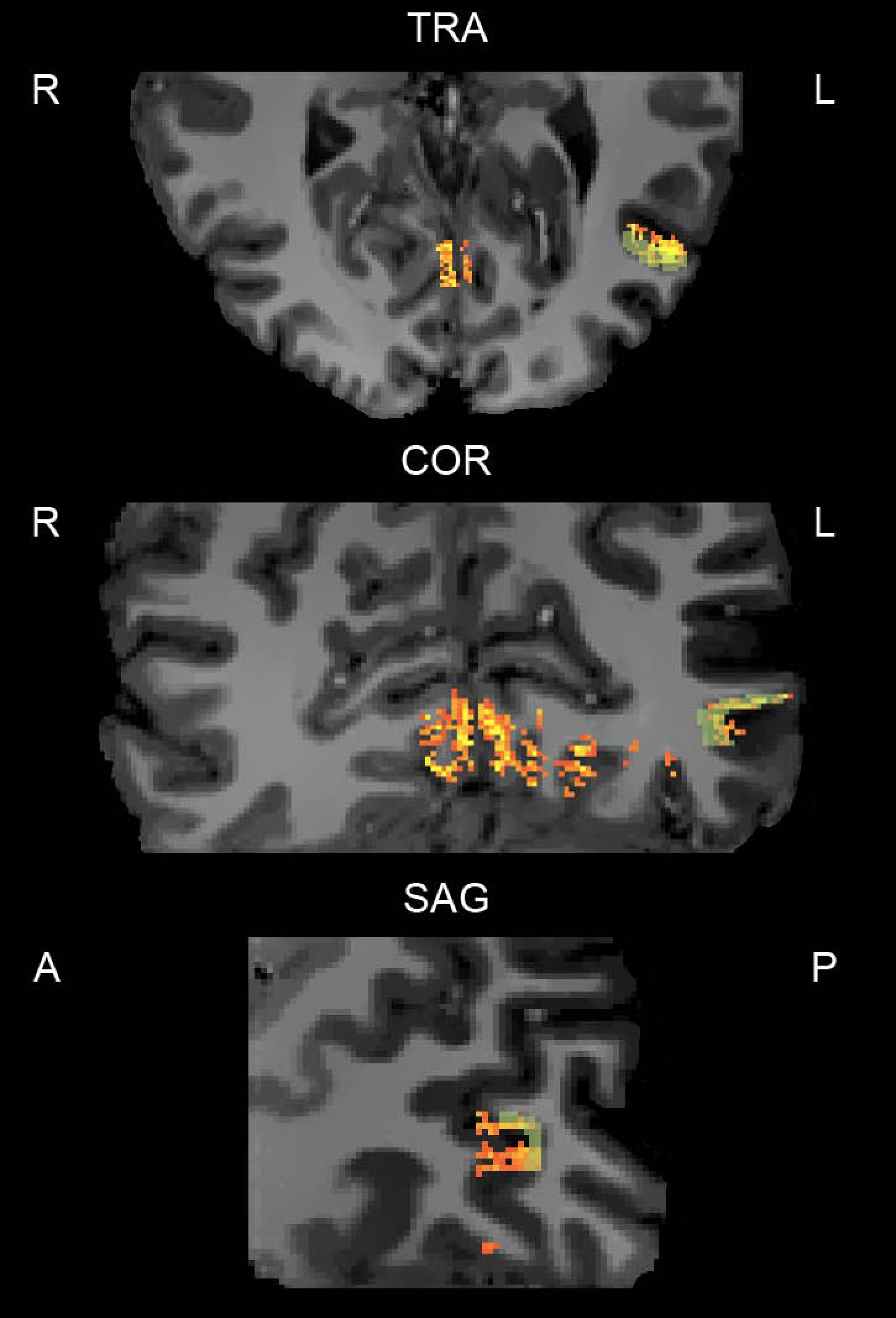

Supplement: Figure S2 — Specificity of the spin-echo sequence used in the axis of motion experiment. Results show the F-map (p<0.01, Bonferroni corrected) comparing the mean of all presented motion directions to baseline exemplary in subject S1. The activity elicited by the stimulation is confined to MT (overlay of the ROI defined from the localization experiment in light green) closely following the cortical gray matter boundary and early visual areas. (TIF) [file pone.0028716.s002.tif]

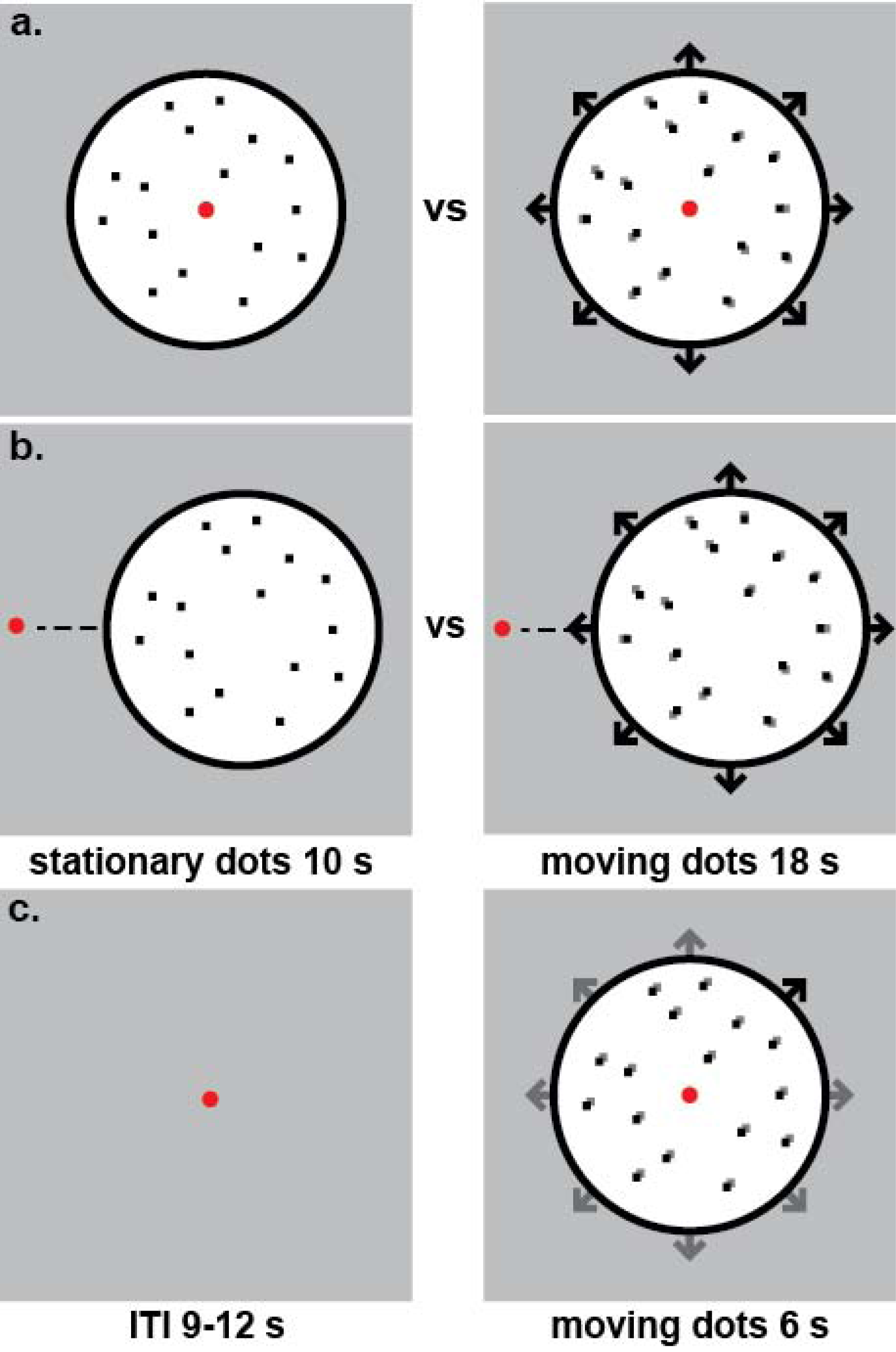

Supplement: Figure S3 — Summary of the stimulation paradigm used. (a) Bilateral moving (18 s) versus stationary (10 s) random dots localizing the hMT+ complex. (b) Ipsilateral moving (18 s) versus stationary (10 s) random dots identifying MST. Peripheral dot patterns were presented in either the left or right visual field while subjects remained fixating on the central fixation spot. (c) Motion direction mapping (6 s) interleaved by a variable inter trial interval. Dots travelled coherently into one 8 randomly presented motion directions (0°, 45°,…, 270°, 315°). (TIF) [file pone.0028716.s003.tif]
